# Biological and Behavioral Responses of *Drosophila melanogaster* to Dietary Sugar and Sucralose

**DOI:** 10.3390/ijms26188951

**Published:** 2025-09-14

**Authors:** Natasha Miranda, Volodymyr V. Tkach, Ana Novo Barros, Ana Martins-Bessa, Isabel Gaivão

**Affiliations:** 1 Animal and Veterinary Research Center (CECAV), Associate Laboratory for Animal and Veterinary Sciences (AL4AnimalS), University of Trás-os-Montes and Alto Douro (UTAD), 5000-801 Vila Real, Portugal; al79678@alunos.utad.pt (N.M.); abessa@utad.pt (A.M.-B.); 2 Centre for the Research and Technology of Agro-Environmental and Biological Sciences (CITAB), University of Trás-os-Montes and Alto Douro (UTAD), 5000-801 Vila Real, Portugal; abarros@utad.pt

**Keywords:** sucrose, sucralose, prolificacy, longevity, DNA damage, *Drosophila*

## Abstract

Sugar and sucralose are frequently used together and separately in human food and beverages, which is the reason why studying their biological action on different organisms is really important. Nevertheless, the effect of highly concentrated sugar diet on male infertility is still under evaluation. The most important is that biological activity of sucralose, a chloroorganic synthetic sweetener, is highly persistent and difficultly altered in the environment, as its influence on the biological activity of other substances has not been completely elucidated yet. For this reason, in this work, sugar and sucralose–sugar mixtures, frequently used in beverages and other food products, influence *Drosophila melanogaster* behavior, longevity, reproductive performance, and genomic integrity is investigated. It has been demonstrated that an increase in sugar concentration promotes biological viability by enhancing prolificacy, lifespan, and locomotor performance. However, this only occurs up to a certain threshold concentration; beyond this, metabolic imbalance occurs. The presence of sucralose in solutions further augments the toxic effect, indicating high genotoxicity of the sweetener at doses over 0.5%, leading to significant DNA alterations and changing the *Drosophila melanogaster* behavior pattern. Therefore, either sugar or sucralose metabolic impact and toxicity is dose-related and their common presence in the solution might lead to the synergetic effect.

## 1. Introduction

The term “sweetener” generally refers to a substance added to food and drinks with purpose of sweetening them [1,2,3,4]. Common sugar or sucrose is the most used sweetener in the world. Nevertheless, the abusive consumption of sugar may lead to complexities like diabetes mellitus type I and II, which is the reason why sugar substitutes are widely used as dietetic sweeteners.

**Figure 1:**
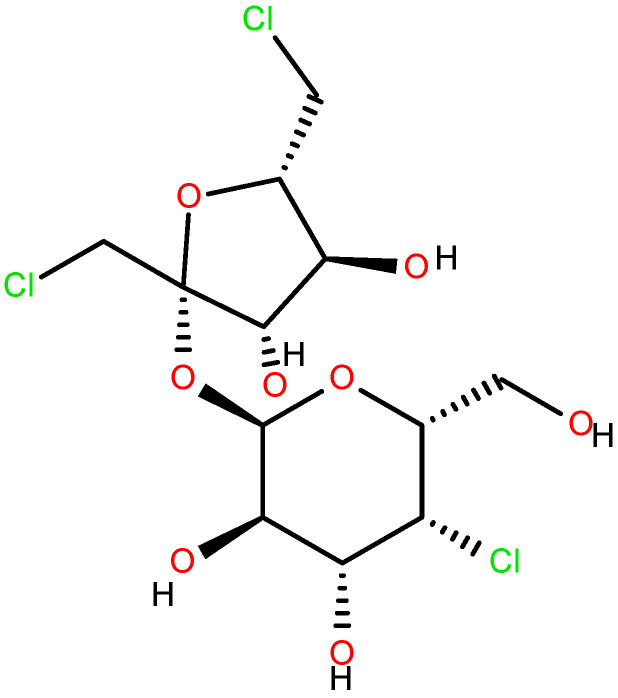
Sucralose.

Sucralose (Figure 1) is one of the most used dietetic sweeteners in Portugal and throughout the European Union in the alimentary and pharmaceutical industries as a flavor corrector [5,6,7,8,9,10]. In the USA, it is also known by its registered trademark, Splenda^®^. In Codex Alimentarius, it is registered as E955. It is a trichloro-substituted derivative of galactosucrose, which has twice the sweetness of saccharin, triple the sweetness of aspartame, and is up to a thousand times as sweet as common sugar. When it comes to its physical properties, free sucralose is a white, shiny, odorless substance that is soluble in water. Contrary to other traditional sweeteners, like aspartame, acesulfame K, saccharin, and stevia, it is a carbohydrate derivative and is the reason why it is extremely sweet, contrarily to the above-mentioned sweeteners.

**Figure 2:**
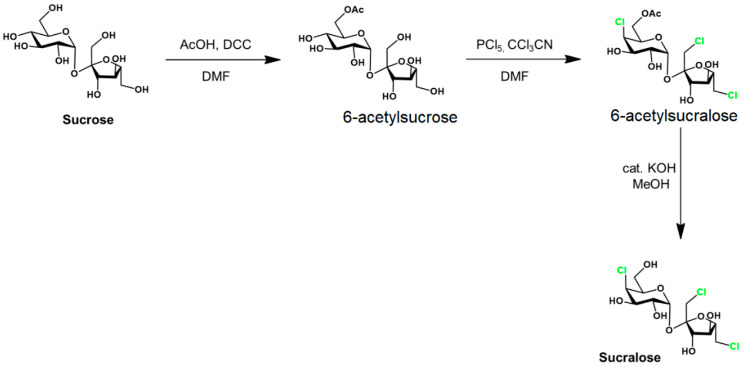
Sucralose synthesis from sucrose. Adapted from [6] with the corresponding permission.

Sucralose is synthetized in food industry from common sugar through several steps (Figure 2).

The acylation in the first stage may also be made by diethylazodicarboxylate. The C4 epimerization is realized in the second stage. This synthesis involves either the toxic reagents (DCC, DAAD, and PCl_5_) or the intermediate (6-acetyl sucralose), as mentioned below.

Despite being considered safe for use by diabetics and athletes, its harmful effects on human health and the environment are still unknown, and some of its negative effects have only begun to be studied now. A recent study involving pregnant and breastfeeding women [11,12,13] confirms that sucralose enters breast milk, causing irreparable damage to the development of the gut microbiota of the human fetus in the last months of pregnancy, as well as in neonates and babies, and is the reason why its safety for use during pregnancy and breastfeeding is still questioned.

**Figure 3:**
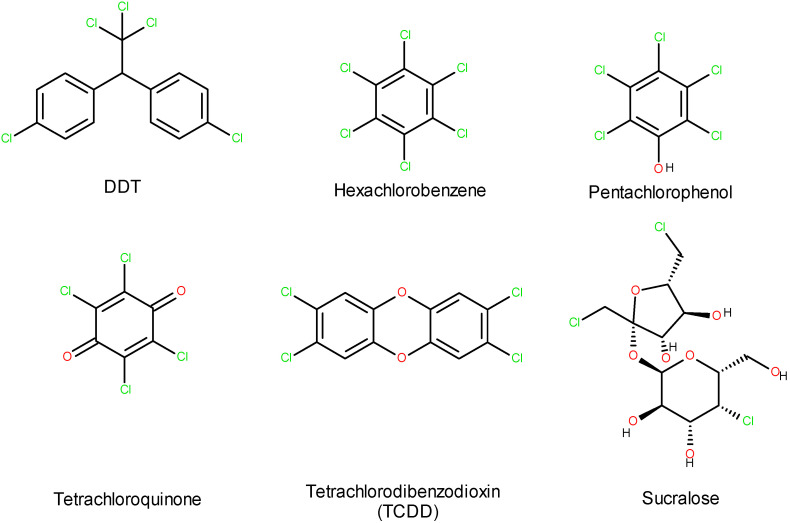
Sucralose among halogen organic compounds.

Moreover, as sucralose is nearly not biodegradable, it accumulates in the environment [14,15,16]. Furthermore, when sucralose decomposes thermally or by some bacterial action, it transforms into toxic compounds such as dioxins and tetrachlorodibenzofurans. It should not be forgotten that sucralose is also part of the group of halogen organic compounds (Figure 3).

Another critical issue yet to be addressed about this compound is the presence of 6-acetyl sucralose, the industrial precursor of the sweetener (Figure 2), in reasonable concentrations in industrial product samples. Furthermore, its appearance in the human intestine is also likely, where it is esterified precisely by the hydroxyl linked to the C6 carbon atom. Recent research has proven either the genotoxicity of 6-acetyl sucralose [17,18,19,20] or the formation of acetylated metabolites in significant concentrations in the human body [21,22].

Toxicological studies of the steric derivative of sucralose proved that the mechanism of its genotoxicity can be considered clastogenic (initiator of breaks in the DNA structure). Even microscopic concentrations of 6-acetyl sucralose, which can be detected in industrial samples and beverages, exceed the safe threshold of 0.15 μg/person/day. The 6-acetyl sucralose ester increases in intestinal epithelial cells, expressing genes linked to inflammation, oxidative stress, and carcinogenesis, including *MT1G* and *SHMT2* [23,24,25].

Another harmful action of the steric derivative is impeding the action of CYP1A2 and CYP2C19, proteins of the cytochrome P450 family, that are responsible for the transformation of various food substances to their more accessible form, which leads to secondary toxic effects [26,27,28,29]. The increase in the genotoxicity of 6-acetyl sucralose concerning sucralose is due to the more significant activity of the secondary organic chloride, linked to the C4 carbon atom, activated by the accepting action of the steric group. The activation of this same atom is also responsible for the mutagenic action of the substance. The sucralose genotoxicity may be realized by either direct interaction with DNA or by indirect induction (generally, by oxidative or metabolic stress, which may lead to secondary oxidative damage). Nevertheless, the investigation of genotoxicity of this sweetener, alone or in the presence of another food components, is still object of an extensive study. A review article [30] describes in detail the state-of-the-art of toxicological effects of sucralose.

Sucralose is frequently used in beverages (soft drinks, fuse teas, dietetic drinks), pharmaceuticals, and dietetic food alongside sugar and other sweeteners (acesulfame K, aspartame, sorbitol, mannitol). This use is aimed to make sweet sensorial properties more expressed in order to enhance the consumer reaction. The same sweeteners are also used in electronic cigarettes, in which they mask the nicotine scent [29].

On the other hand, as for now, few to no studies are known to describe the sucralose biological activity, including genotoxicity, in the presence of other substances, including natural and synthetic sweeteners [31,32,33,34,35,36,37,38,39,40], food contaminants, and food contact substances, showing the influence of sucralose mixtures with other substances on oxidative, genomic, and environmental stress. The cases of synergism in oxidative and genomic stress of sucralose and other compounds (including aspartame and benzo[a]pyrene) have already been observed [31,32,33,34,35]. For this reason, the influence of sucralose on the biological impact of these substances is an object of an extensive study.

In this aspect, *D. melanogaster* is suitable for biological, behavioral, and genotoxicity essays of sucralose mixtures with other substances [41,42,43,44,45,46,47,48,49,50]. It is a widely used model organism in genotoxicity assays due to its unique genetic and biological characteristics due to six main reasons:

- Shares a substantial proportion of its genome with humans, with approximately 60% of human disease-associated genes having identifiable homologs. Its fully sequenced and extensively annotated genome makes it a valuable model for assessing the impact of mutagens on specific genes and regulatory pathways that are conserved across species, including humans.
- It has a short life cycle (approximately 10–12 days at 25 °C) and produces large numbers of offspring, making it particularly suitable for large-scale genetic screens and statistical analyses of mutagenic effects across generations. These features are essential for the efficient and cost-effective assessment of genotoxic risk.
- There is an extensive collection of mutant lines, which enables the study of a wide range of biological conditions and responses. This genetic diversity allows researchers to investigate the effects of genotoxic agents under different genetic backgrounds, contributing to a more comprehensive understanding of mechanisms involved in DNA damage and repair.
- Compared to mammalian models, the use of *D. melanogaster* raises fewer ethical concerns and is significantly more cost-effective, and its small size and ease of handling make it well suited for high-throughput screening of chemicals, radiation, and environmental pollutants for genotoxic potential.
- Despite frequent application of *Caenorhabditis elegans* as a model for food additive safety genotoxicity tests, *Drosophila* is a much more versatile tool, capable of being applied to the genotoxicity test of sucralose as both a food additive and an environmental contaminant.
- It has a simple and well-characterized gut microbiota that can be experimentally manipulated, allowing the investigation of how diet, environmental agents, or genetic factors influence microbial composition and host physiology.

In this study, *D. melanogaster* was used to perform biological, behavioral, and genotoxicity assays with different concentrations of sugar, both alone and in combination with sucralose, administered chronically through the diet throughout larval development. The aim was to characterize for the first time the concentration-dependent effects of each compound individually and to explore potential synergistic interactions between them, leading to DNA damage, as well as behavioral and biological changes, resulting from the genomic stress. Such interactions could result in enhanced metabolic dysregulation and increased toxicity, thereby providing insight into their combined biological impact.

## 2. Results

### 2.1. Proficiency

**Figure 4:**
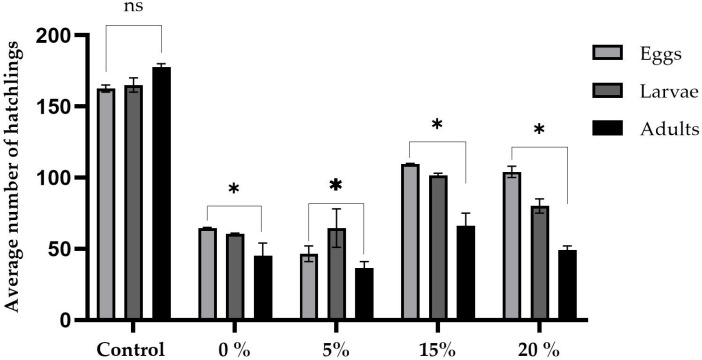
Prolificacy assay showing the average number of eggs, larvae, and adult flies obtained from *D. melanogaster* cultures exposed to different dietary concentrations of sugar (sucrose): 0%, 5%, 15%, and 20%, compared to the control group (10% sucrose, standard medium). Data are presented as the mean ± standard deviation. * *p* < 0.05, ns *p* > 0.05.

**Figure 5:**
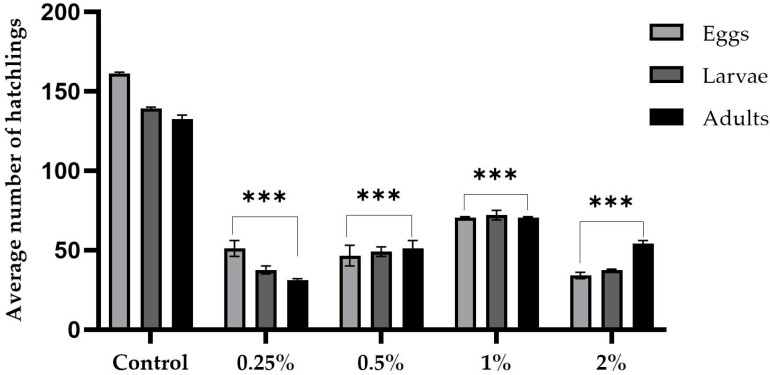
Prolificacy assay showing the average number of eggs, larvae, and adult flies obtained from *D. melanogaster* cultures exposed to dietary sucralose at concentrations of 0.25%, 0.5%, 1%, and 2%, compared to the control group (10% sucrose only). Data are presented as the mean ± standard deviation. *** *p* < 0.001.

Figure 4 and Figure 5 summarize the prolificacy results for the sugar and sucralose–sugar treatments and their respective concentrations and control. Five males and five virgin females were used per tube. A total of 15 tubes for each concentration, with three independent replicates.

An analysis of the reproductive capacity of *Drosophila melanogaster* exposed to different concentrations of sucrose revealed statistically significant differences among the three evaluated stages: eggs, larvae, and adults (Figure 4). The sucrose control group produced an average of 162 eggs, 165 larvae, and 177 adults. The absence of sucrose drastically reduced these values to an average of 64 eggs, 60 larvae, and 45 adults. These values were significantly different from those of the control group (*p* < 0.0001 at all stages). The 5% sucrose group produced an average of 46 eggs, 64 larvae, and 36 adults. These values were also significantly lower than those of the control group (*p* < 0.001). In contrast, 15% of the sucrose group had fecundity similar to that of the control group, with averages of 109.0 eggs, 101 larvae, and 66 adults (*p* > 0.05). However, the 20% concentration caused a significant reduction: 104 eggs, 80 larvae, and 49 adults (*p* < 0.001).

Adding sucralose to a medium containing 10% sucrose negatively impacted prolificacy in a dose-dependent manner (Figure 2). The control group had an average of 161 eggs, 139 larvae, and 132 adults. However, 0.25% sucralose concentration resulted in a significant reduction, with an average of 51 eggs, 37 larvae, and 31 adults (*p* < 0.001). At 0.5%, the values were 46 eggs, 49 larvae, and 51 adults (*p* < 0.001). Concentrations of 1% and 2% further aggravated the effects. There were 70 eggs, 72 larvae, and 70 adults at 1%, and 34 eggs, 37 larvae, and 54 adults at 2% (all *p*-values < 0.0001).

### 2.2. Longevity

**Figure 6:**
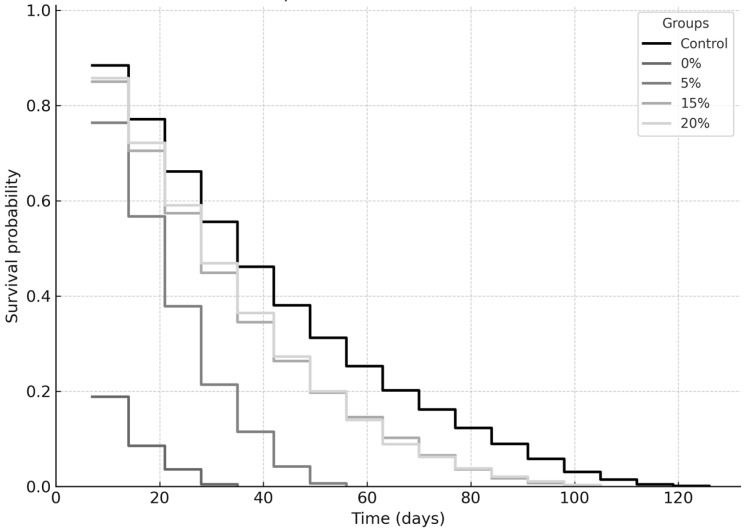
Kaplan–Meier survival curves for *Drosophila melanogaster* containing different concentrations of sucrose: 0%, 5%, 15%, and 20%, compared to the control group (10% sucrose). The survival probability is plotted over time (days).

**Figure 7:**
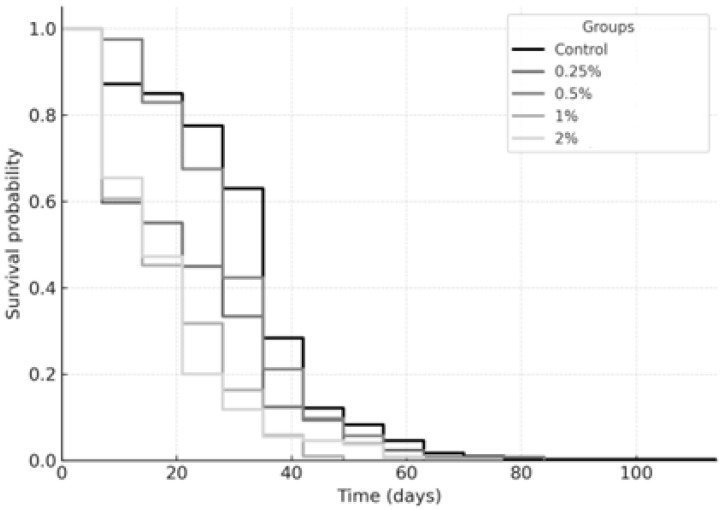
Kaplan–Meier survival curves for *Drosophila melanogaster* containing sucralose at concentrations of 0.25%, 0.5%, 1%, and 2%, compared to the control group (10% sucrose only). The survival probability is plotted over time (days).

Figure 6 and Figure 7 summarize the longevity average (Kaplan–Meier survival curves), results for the sugar and sucralose–sugar treatments and their respective concentrations and control. A total of 15 tubes for each concentration, with three independent replicates.

The Kaplan–Meier survival analysis revealed that sucralose and sugar supplementation both significantly reduced the survival of *Drosophila melanogaster*. Sucralose (Figure 4) was tested at the following concentrations: control, 0.25%, 0.5%, 1%, and 2%. The survival curves showed a clear, concentration-dependent decline in longevity. Flies exposed to 0.25% sucralose (log-rank, *p* = 0.041), 0.5% (*p* = 0.008), 1% (*p* < 0.001), or 2% (*p* < 0.0001) exhibited progressively reduced survival probabilities compared with control. The strongest effect was observed at 2%, indicating that higher concentrations of the non-caloric sweetener markedly accelerated mortality. For sugar (control, 0%, 5%, 15%, 20%), as shown in Figure 3, a similar dose–response relationship was observed. All tested concentrations significantly shortened lifespan compared to the control group: 0% (*p* = 0.037), 5% (*p* = 0.012), 15% (*p* < 0.001), and 20% (*p* < 0.0001). The most pronounced reduction occurred at 20% sugar, confirming the deleterious effect of high-calorie sweetener concentrations. Taken together, these results demonstrate that both caloric (sugar) and non-caloric (sucralose) sweeteners modulate survival dynamics in fruit flies, with increasing concentrations consistently associated with reduced longevity.

### 2.3. Negative Geotaxis Test

**Figure 8:**
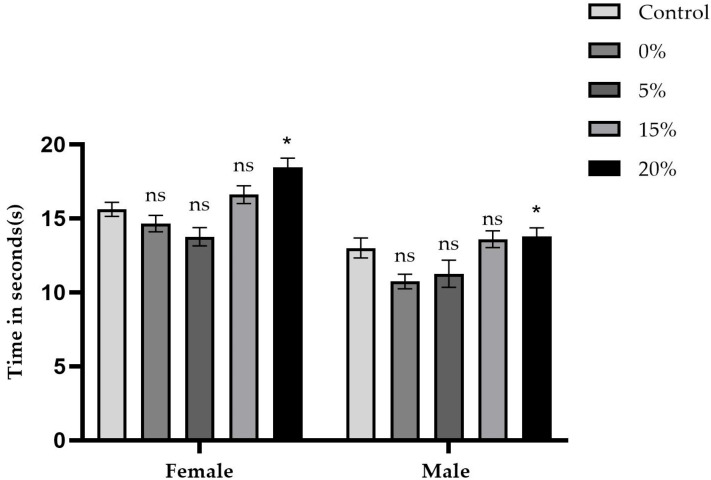
Results of the negative geotaxis assay in *D. melanogaster* cultured on media containing sucrose at concentrations of 0%, 5%, 15%, and 20% compared to the control group (10% sucrose). The average climbing time (in seconds) ± SD is presented separately for males and females. An asterisk (*) indicates a statistically significant difference (*p* < 0.05) compared to the control group. ‘ns’ indicates no statistically significant difference.

**Figure 9:**
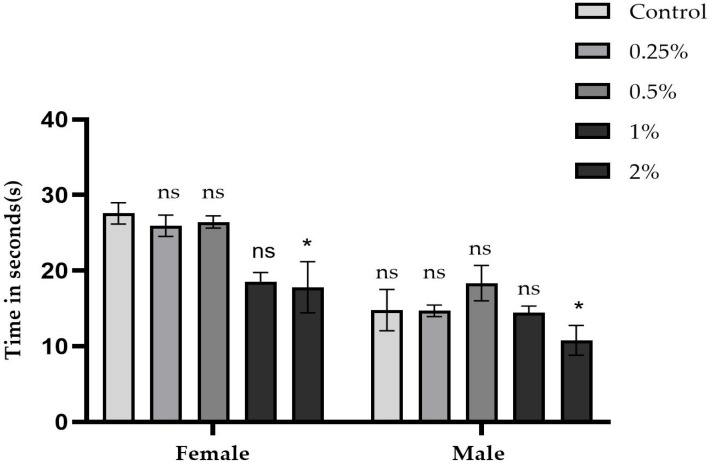
Results of the negative geotaxis assay in *D. melanogaster* cultured on media containing sucralose at concentrations of 0.25%, 0.5%, 1%, and 2% compared to the control group (10% sucrose only). Average climbing time (in seconds) ± SD is shown separately for males and females. Data are presented as the mean ± standard deviation. * *p* < 0.05, ns *p* > 0.05.

Figure 8 and Figure 9 summarize the average time, in seconds, that five male and five female flies took to climb tubes marked at 8 cm, as well as the respective concentrations of the sugar, sucralose–sugar, and control treatments. A total of 15 tubes were used for each concentration, with three independent replicates.

Vertical locomotor performance (Figure 8), as measured by the negative geotaxis test, was significantly impacted by the concentrations of both sucrose and sucralose. The sucrose control group had average climbing times of 12.9 s for males and 15.6 s for females. Removing sucrose entirely (0%) decreased the times to 10.7 s (males) and 14.6 s (females), *p* < 0.0001. At 5%, the times were similar to those of the control group (11.7 and 13.7 s, respectively; *p* > 0.05). However, at 15% and 20% concentrations, climbing times increased significantly: 13.5 s (males) and 16.6 s, and 18.6 s (females) (*p* < 0.01).

For groups exposed to sucralose (Figure 9), control times were 14.7 s (males) and 27.7 s (females). The 0.25% (14.7 s for males and 25.9 s for females) and 0.5% (18.4 s for males and 26 s for females) concentrations did not statistically differ from the control (*p* > 0.05). At 1% and 2% concentrations, however, the times were 11.7–14.4 s (males) and 17.8–18.5 s (females), respectively. For the 1% concentration, the difference was statistically significant (*p* < 0.05), while for the 2% concentration the difference was also significant (*p* < 0.05).

### 2.4. Exploration Test

**Figure 10:**
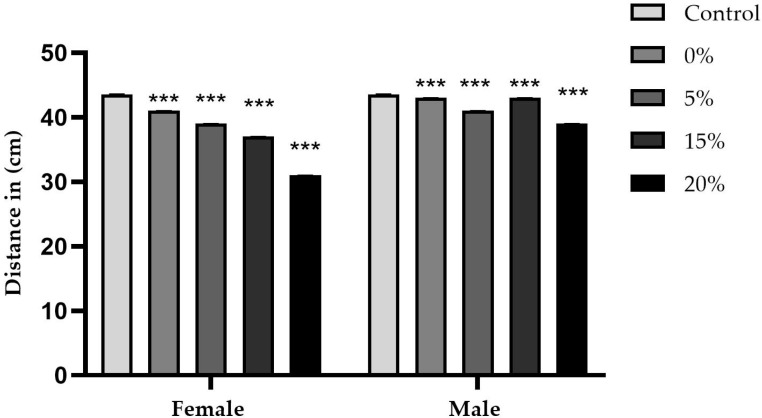
Exploration test from cultures containing sugar (sucrose) at concentrations of 0%, 5%, 15%, and 20%, as well as control data. Data are presented as the average distance (in cm) traveled in one minute in a Petri dish, separated by sex and presented as mean ± SD. “An asterisk (***) indicates a statistically significant difference (*p* < 0.001) compared to the control group”.

**Figure 11:**
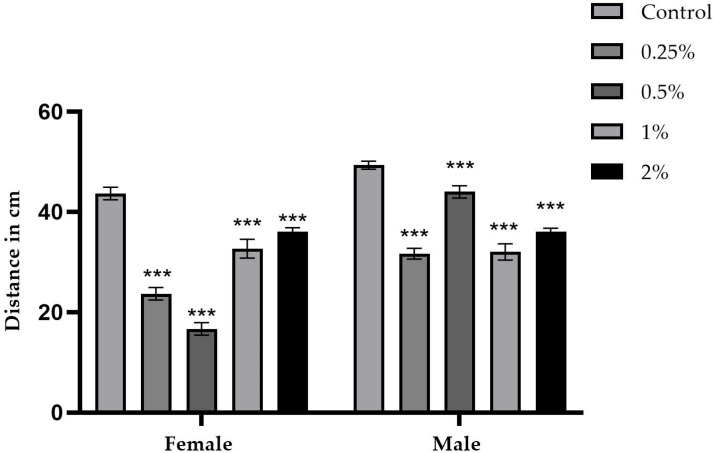
Exploration test from cultures containing sucralose at concentrations of 0.25%, 0.5%, 1%, and 2%, as well as control data. Data are presented as the mean distance in cm traveled in one minute in a Petri dish. Data are presented as the mean ± standard deviation. *** *p* < 0.001.

Figure 10 and Figure 11 summarize the average distance traveled by five male and five female flies per cm explored in 1 min in a Petri dish, as well as the respective concentrations of the sugar and sucralose–sugar treatments and the control. A total of 15 tubes for each concentration, with three independent replicates, were performed.

Exploratory behavior was assessed by measuring the average distance traveled in one minute, in square centimeters. In the sucrose control group (Figure 10), males and females traveled an average of 43.5 cm per min. The absence of sucrose slightly reduced these distances to 41 cm for males and 43 cm for females (*p* < 0.0001). At 5% concentration, performance remained the same; however, concentrations of 15% and 20% resulted in significant reductions in females: 37.5 and 31 cm, respectively (*p* < 0.005). Meanwhile, males remained between 43 and 39 cm, respectively.

In the sucralose-treated groups (Figure 11), controls covered an average distance of 49 cm for males and 43 cm for females. At the 0.25% concentration, males covered an average distance of 31 cm and females 23 cm. At the 0.5% concentration, males covered an average distance of 44 cm and females 16 cm, compared to the control group. The 0.25% and 0.5% concentrations showed statistically significant differences (*p* < 0.001). In the 1% and 2% concentrations, values decreased to 32 cm, compared to the control group. The difference was statistically significant in all tested sucralose concentrations, with *p* < 0.001.

### 2.5. Comet Assay Results

**Figure 12:**
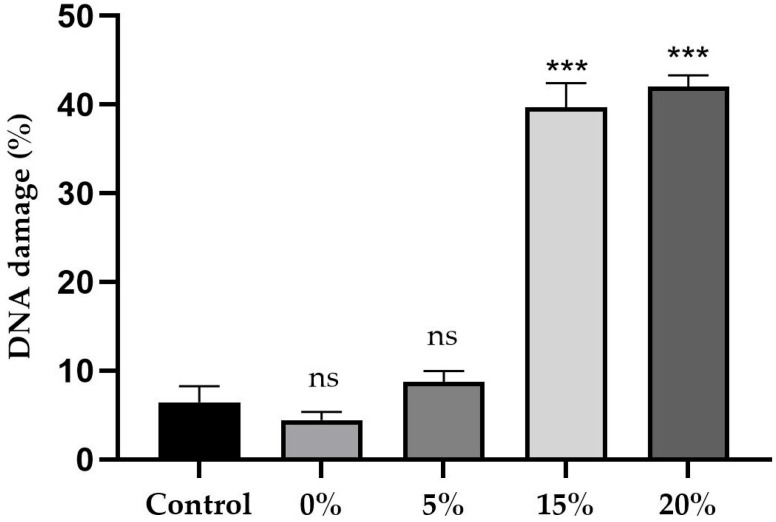
The results of the in vivo comet assay show the percentage of DNA damage in the neuroblasts of larval brains in cultures with 0%, 5%, 15% and 20% sucrose, alongside control data (10%). Data are presented as the mean ± standard deviation (SD). An asterisk (***) indicates a statistically significant difference (*p* < 0.001) compared to the control group. ‘ns’ indicates no statistically significant difference.

**Figure 13:**
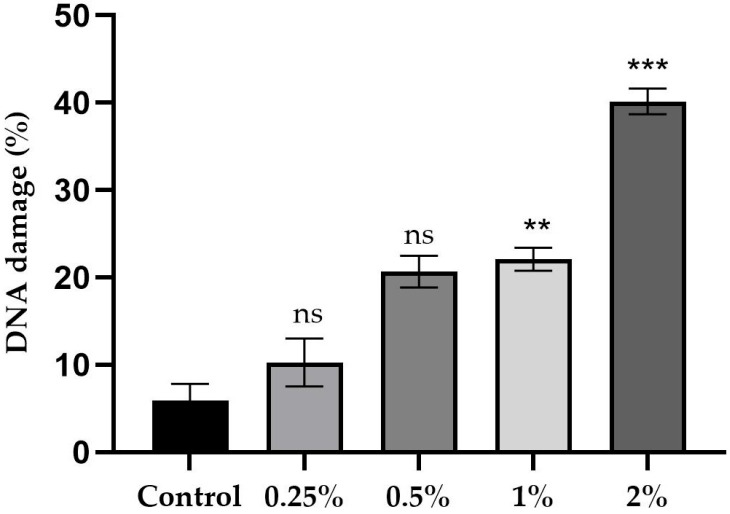
Comet assay results show the percentage of DNA damage in neuroblasts from *D. melanogaster* larval brains in cultures with 0.25%, 0.5%, 1%, and 2% sucralose, along with control data. Data are presented as mean ± standard deviation (SD). An asterisk (** and ***) indicates a statistically significant difference (*p* < 0.001) compared to the control group. ‘ns’ indicates no statistically significant difference.

Figure 12 and Figure 13 summarize the results of the in vivo comet assay, showing the percentage of DNA damage in brain neuroblasts of third-stage larvae. The figures show neuroblasts exposed to a culture medium containing concentrations of sugar and sucralose–sugar, as well as the respective control groups. A total of 15 tubes were used, with three independent replicates.

Assessments of DNA damage in larval neuroblasts revealed significant genotoxic effects related to sucrose and sucralose concentrations. The sucrose control group (Figure 12) exhibited 6.5% DNA damage in the comet tail assay. Sucrose deprivation (0%) resulted in 4.6% damage (*p* < 0.0001). At 5%, the value was 8.7% (*p* > 0.05). However, at concentrations of 15% and 20%, the values increased to 39.6% and 42%, respectively (*p* < 0.01), indicating significant genetic material damage.

As for sucralose (Figure 13), the control group showed 5.9% damage. Concentrations of 0.25% and 0.5% resulted in 10.5% and 20% damage, respectively (*p* > 0.05). However, damage increased substantially to 22% and 40%, respectively, at concentrations of 1% and 2% (*p* < 0.001), indicating sucralose’s high genotoxicity at concentrations above 0.5%.

## 3. Discussion

This study reveals the significant effects of different sucrose and sucralose concentrations on various biological and behavioral parameters of *D. melanogaster*, including prolificacy, longevity, locomotion, exploratory behavior, and DNA integrity. These findings corroborate and in some respects contrast with previously reported data, providing a more comprehensive understanding of the mechanisms underlying metabolic and genotoxic toxicity induced by natural and artificial sweeteners.

With regard to prolificacy, the data show that the absence (0% and 5%) or excess of sucrose (20%) significantly reduces the number of eggs, larvae, and adults, with peak performance observed at 10–15% sucrose. This dose-dependent pattern is consistent with the findings of Skorupa et al. [51], who demonstrated that the composition of the diet directly influences feeding behavior, obesity, and fecundity in *D. melanogaster*. Similarly, Piper et al. [52] showed that excessively high-calorie or low-calorie diets negatively affect energy balance and reproduction. Notably, sucralose induced a more abrupt and severe drop in prolificacy than total sucrose deprivation, suggesting that its impact extends beyond caloric imbalance to potentially interfere with key reproductive or metabolic pathways. This pattern is consistent with the observations of Pepino [53], who described the adverse metabolic effects of artificial sweeteners, and of Rogers and Appleton [54], who reported that sucralose disrupts the gut microbiota and may impair reproductive function in non-mammalian models.

Longevity analysis revealed that both sucrose deprivation and excess decreased the mean lifespan of individuals, with the most significant impact observed in the absence of sucrose. These findings align with those of Partridge et al. [55], who linked unbalanced diets to a shorter lifespan due to increased oxidative stress and metabolic dysfunction. Katewa and Kapahi [56] also demonstrated that mild caloric restriction can be beneficial, whereas severe deficiency can compromise survival. This explains the adverse outcomes observed in groups without sucrose supplementation. The progressive reduction in lifespan with increasing sucralose concentrations, even at 0.5%, raises concerns about its cumulative toxicity, possibly linked to chronic disruption of gut microbiota and metabolic homeostasis. Data from Abou-Donia et al. [57] on rats are consistent with these findings, showing that sucralose alters gut microbiome and induces chronic metabolic inflammation impacting lifespan.

Both negative geotaxis and exploration assays reveal sex-specific and dose-dependent locomotor deficits, especially under sucralose exposure. These deficits are likely to reflect underlying neurometabolic disruption, consistent with previous reports of dopamine impairment and oxidative stress caused by excessive sugar intake or artificial sweeteners. Females generally exhibited greater sensitivity to sucralose exposure, with significant performance impairment occurring at 0.25% exposure. These results are consistent with those of Gargano et al. [58], who developed the RING method to detect early locomotor decline in *Drosophila* and demonstrated that dietary changes significantly affect motor function. Musselman et al. [59] also demonstrated that high-sugar diets reduce dopamine levels in *Drosophila*, thereby impairing locomotor and exploratory activity, which explains the decreased climbing times and exploratory distances observed in this study.

The comet assay revealed a significant increase in DNA strand breaks in larval neuroblasts exposed to high concentrations of sucrose (≥15%) or any concentration of sucralose (starting at 0.5%). The observed genotoxicity with elevated sucrose may be linked to increased production of reactive oxygen species (ROS), as described by Diniz et al. [60], who reported on oxidative stress in rats on high-sugar diets. The DNA damage observed, particularly with sucralose exposure, may result from oxidative lesions and impaired repair mechanisms. Studies from Schiffman and Rother [61] have shown modulation of key DNA repair genes (e.g., APTX, EID1), supporting a mechanistic basis for sucralose-induced genotoxicity. Bornemann et al. [62] recently reinforced these findings by demonstrating that chronic sucralose exposure in human colon cells results in elevated oxidative stress and direct DNA strand breakage, thus supporting the genotoxicity observed in *Drosophila*.

Other direct and indirect genotoxicity issues for sucralose have been conducted recently [38,63,64,65,66,67]. In a 2019 study [38], in *Cyprinus carpio*, chronic exposure led to DNA damage, increased ROS, and apoptosis in blood cells.

Other studies have raised more serious concerns. For instance, ref. [64] reported that thermal degradation products of sucralose are toxic and capable of reacting with DNA. This study, which evaluated sucralose’s cyto-, geno-, and immunotoxicity through in vitro and in silico methods, found a non-selective reduction in CD4+ and CD8+ lymphocyte populations, along with dose-dependent DNA alterations and chromosomal structural changes in lymphocytes. These effects were associated with the modulation of genes such as *MAPK8*, *APTX*, and *EID1*. Studies on *Allium cepa* provided further evidence of sucralose’s genotoxic potential. Exposure to aspartame, sorbitol, and sucralose resulted in various chromosomal abnormalities [65], most notably micronuclei formation during interphase and mitosis in root tip cells—a key indicator of mutagenesis. A follow-up study [66] that examined sucralose, aspartame, and their combination revealed synergistic genotoxic effects in the same plant model. Animal studies have also supported these findings. One of the earliest detailed investigations [67] exposed male Swiss mice prenatally to sucralose, observing a dose-dependent increase in hematopoietic neoplasms when sucralose was administered along with its hydrolysis product 6-CF at concentrations of 2000 ppm and 16,000 ppm. These findings suggest that sucralose may influence epithelial and glandular tissues in both benign and malignant processes. These studies are among the few known investigations of sucralose genotoxicity in the presence of other sweeteners and on the plant organisms, which becomes highly important, due to the chloroorganic nature of sucralose, similar to herbicides and insecticides.

Therefore, the present study on *D. melanogaster* confirms the general trend of assessing sucralose genotoxicity in the presence of sugar and other sweeteners and food contact substances, which deserves to be studied in depth. This becomes even more important due to the tendency for sucralose accumulation in the environment.

In summary, our findings suggest that moderate doses of sucrose support normal physiological parameters in *D. melanogaster*, whereas deprivation or excess has harmful effects. In contrast, sucralose exhibited significant toxicity even at low concentrations, impairing reproductive viability, lifespan, motor function, and genomic integrity, with more pronounced effects observed in females. Given the widespread presence of sucralose in aquatic systems and its apparent bioaccumulation potential, these findings in *D. melanogaster*—a model organism with conserved metabolic and genetic pathways—call for urgent reevaluation of regulatory thresholds for artificial sweeteners in food and the environment. Future studies should investigate the molecular mechanisms underlying sucralose toxicity, including alterations in antioxidant gene expression, DNA repair pathways, and neuroendocrine signaling, as well as potential transgenerational effects. More profound studies, including RT, qPCR, ROS quantification (which may be realized both chemically and electrochemically), providing explicit mechanisms of direct or indirect sucralose genotoxicity with and without the presence of sugar and other sweeteners are also important and will be exposed in our further studies.

## 4. Materials and Methods

### 4.1. Strains

The Oregon-K (OK) strain was used in this study. It is a wild-type line proficient in all major DNA repair pathways, including homologous recombination (HR), classical non-homologous end joining (c-NHEJ), and microhomology-mediated end joining (MMEJ). Additionally, this strain has been biochemically characterized and shown to have low antioxidant enzyme activity, which makes it particularly sensitive for studies involving oxidative damage [68,69].

### 4.2. Culture Medium Conditions and Sample Preparation with Drosophila

All flies were maintained in a climate-controlled chamber at 24 °C with a 12:12 h light–dark cycle. To maintain the population, newly eclosed adults were transferred to glass vials containing standard culture medium. The composition of this medium is detailed in Table 1. Vials were sealed with cotton plugs to allow airflow and prevent contamination.

Flies were divided into experimental groups according to the treatment regimen. The control group consisted of flies maintained on standard medium (containing 10% sugar), treatment groups included flies exposed to sugar concentrations of 0%, 5%, 15%, and 20%, as well as flies maintained on a culture medium with 10% sugar supplemented with sucralose at concentrations of 0.25%, 0.5%, 1%, 2%, and 10%. Virgin males and females aged between 0 and 3 days were selected for the experiments. For each group, five mating vials were prepared, each containing ten virgin males and ten virgin females. Males in the treatment groups were exposed to the respective sugar and sucralose concentrations for 2 to 3 days under standard conditions (24 °C). Immediately following exposure, males were introduced to females for mating.

### 4.3. Prolificacy Evaluation

Prolificacy was assessed using a standard culture medium (described in Table 1) supplemented with 0.5% activated charcoal (Sigma-Aldrich, St. Louis, MO, USA). This medium was added to both treatment and control vials as previously described. Following exposure and mating, the numbers of eggs, larvae, and viable adults were recorded. Activated charcoal was added to darken the culture medium, providing contrast that facilitated accurate eggs counting [70,71]. All assays were conducted in triplicate, with three independent replicates of 15 mating vials per group.

### 4.4. Longevity Assay

After offspring counting, individuals from each experimental group (control and treatment with sugar or sucralose) were transferred to three replicate vials per group. Each vial consisted of a 200 mL glass container with approximately 25 mL of culture medium. Flies were transferred to fresh vials every seven to nine days to maintain food quality and avoid generational overlap. Mortality was recorded at each transfer until all individuals in each group had died. Longevity was calculated as the number of days from hatching to death for each fly [72].

### 4.5. Open Field Exploration Test (Evaluation of Exploratory)

Approximately five adult flies, separated by sex, from both treatment and control groups (as described in Section 2.3), were introduced into a transparent 10 cm diameter circular arena marked with quadrants of 1 cm. Following exposure to sugar and sucralose treatments, the flies’ spontaneous locomotion was recorded on video. Their movement trajectories were then analyzed by measuring the total distance traveled over a 1 min period [73].

### 4.6. Negative Geotaxis Test (Locomotion)

Groups of ten adult flies, separated by sex, from both treatment and control groups (as described in Section 2.3), were placed in glass transparent tubes, 15 cm in height. The tubes were held vertically and tapped firmly three times against a flat surface. The time taken for each fly to climb to a height of 8 cm was recorded in seconds, and the average climbing speed was calculated as a percentage per second. Three trials were conducted per group, with a one-minute interval between each trial [74,75].

### 4.7. Comet Assay

The comet assay was carried out to assess DNA strand breaks, alkali-labile sites, and general genomic integrity in neuroblasts. Approximately 2 to 3 third-stage larvae were collected from each experimental group and the neuroblasts were isolated by dissection using a drop of ringer’s solution (150 mM NaCl, 35 mM KCl, and 2 mM CaCl, adjust the pH to 6.5 with NaOH). The extracted tissues were gently dissociated using forceps into a 1.5 mL microtube with about 100 uL of insect ringer’s solution (130 mM NaCl, 35 mM KCl, 2 mM CaCl_2_; pH adjusted to 6.5 with NaOH) and subsequently centrifuged at 300× *g* for 5 min. After centrifugation, the supernatant was discarded, and 140 µL of 1% low-melting-point agarose (Pronadisa Micro and Molecular Biology, Madrid, Spain), in PBS, maintained at 37 °C, was added to each microtube. Two 70 µL drops of the resulting cell–agarose suspension were then placed onto pre-coated microscope slides with 1% normal-melting-point agarose (Sigma-Aldrich, St. Louis, MO, USA). Each drop was immediately covered with an 18 × 18 mm coverslip and left to solidify at 4 °C for five min. The coverslips were then carefully removed. The slides were then immersed in a lysis buffer (2.5 M NaCl, 0.1 M EDTA, 10 mM Tris-base, 1% Triton X-100, pH 10) for 1 h at 4 °C. Following lysis, the slides were transferred to an electrophoresis buffer (0.3 M NaOH, 1 mM EDTA, pH 12.6) and incubated for 30 min at 4 °C to allow DNA unwinding. Electrophoresis was then performed at 25 V/cm and 300 mA for 20 min at 4 °C using a standard electrophoresis unit. After electrophoresis, the slides were neutralized by sequential washes with 1× PBS and distilled water for 10 min each at 4 °C. DNA was stained with 20 μL of DAPI solution (1 μg/mL in H_2_O) per gel. One hundred nuclei per gel were scored visually using a fluorescence microscope (Olympus BX41, 40× magnification, by Olympus, Hachioji, Japan). Comets were visually categorized into five damage classes based on tail intensity, with scores ranging from 0 (no damage) to 4 (maximum damage) [76]. The cumulative score for each gel was calculated by summing the scores of the 100 comets, resulting in a scale from 0 to 400 arbitrary units (AU). DNA damage was expressed as the percentage of DNA in the tail, calculated by dividing the AU value by 4, as described by Azqueta et al., 2011. The scoring was performed blindly to ensure an unbiased assessment [77].

### 4.8. Data Evaluation and Statistical Analysis

The DNA damage data obtained through the comet assay (expressed as the percentage of DNA in the comet tail) were analyzed using a one-way analysis of variance (ANOVA). Statistical comparisons were performed only on the percentage of DNA damage.

Graphical representation organized in bars, showing the mean distance traveled by males and females in seconds and centimeters were used to present data from the negative geotaxis test and fly exploration. Prolificacy was assessed based on the number of adults, larvae, and hatched eggs. Quantitative data are presented as the mean ± standard deviation (SD). An independent samples *t*-test was performed to determine statistically significant differences between groups. This was done for the negative geotaxis test and for comparisons between adults, larvae, and hatched eggs. To assess longevity among *Drosophila* groups, survival curves were calculated using the Kaplan–Meier method with 95% confidence intervals. The log-rank (Mantel–Cox) test was used to compare survival curves between groups and to assess paired differences in survival distributions. All statistical analyses were performed using GraphPad Prism software (version 9.0, GraphPad Software, San Diego, CA, USA). All results are expressed as the mean ± standard deviation (SD) from three independent replicates.

## 5. Conclusions

The present investigation demonstrates that the effects of sucrose and sucralose on the physiology, behavior, and genome of *D. melanogaster* are complex, dose-dependent, and, in some cases, synergistic. Moreover, the synergism between the two can also be observed. Moderate sucrose concentrations supported biological viability, enhancing reproductive output, lifespan, and locomotor function. In contrast, both deprivation and excessive intake of sucrose elicited detrimental outcomes, reflecting underlying metabolic imbalances. In contrast, deprivation or excess of this carbohydrate led to significant impairments indicative of metabolic imbalance. Sucralose, however, exhibited marked toxicity even at low concentrations, leading to reduced prolificacy and lifespan, impaired exploratory behavior, and significant genomic damage as evidenced by comet assay results. Given the increasing prevalence of artificial sweeteners in human diets and their accumulation in the environment, these findings raise important concerns regarding their long-term biological safety. Moreover, they highlight the value of *D. melanogaster* as a sensitive and robust model for assessing dietary toxicity and genotoxic risk, providing insights that may support future regulatory assessments and public health policies.

## Figures and Tables

**Table 1:** Constituents of the standard culture medium.

Ingredients	Amount (Per Liter in Distilled Water)
Sucrose	100 g
Agar-agar	12 g
Inactive yeast	100 g
Propionic acid	5 mL

## Data Availability

All the new data obtained in this research may be accessed and explained by contacting the correspondent authors.
